# Effect of maternal lactoferrin supplementation on iron contents and anti-oxidant capacity in Dahe black Pig neonates

**DOI:** 10.3389/fvets.2022.1034084

**Published:** 2022-10-31

**Authors:** Chunyong Zhang, Cenxi Li, Xiaokun Xing, Peng Ji, Meiquan Li, Hongbin Pan, Rongfu Guo, Qingcong An

**Affiliations:** ^1^Yunnan Provincial Key Laboratory of Animal Nutrition and Feed Science, Faculty of Animal Science and Technology, Yunnan Agricultural University, Kunming, China; ^2^Jianshui County Animal Husbandry Technology Extension Station, Honghe, China; ^3^College of Agriculture and Life Sciences, Kunming University, Kunming, China

**Keywords:** lactoferrin, iron content, Dahe black pig, gene expression level, sows, neonates

## Abstract

Iron levels are closely related to animals' growth performance and anti-oxidant function. Lactoferrin (LF) is an iron-binding glycoprotein, which can promote the absorption of iron and regulate immune function. This study aimed to clarify the effect of maternal LF supplementation on the iron metabolism of Dahe piglets. Sixty sows (Dahe black, parity 3-4, no significant differences in body weight) were randomly assigned to five groups: control (basal diet with no iron supplementation), supplemented 100 (LF1 group), 200 (LF2 group), or 300 (LF3 group) mg LF/kg in the basal diet, and the basal diet supplemented with 100 (Fe-Gly group) mg Fe/kg as ferrous glycine (Fe-Gly). The serum anti-oxidant parameters of the sows and neonatal piglets were determined. The iron contents, anti-oxidant gene expression levels, and Fe-acquisition genes were detected in the liver, heart, spleen, and other neonatal organs. The results indicated that (1) the LF3 group of sows had the highest serum and colostrum iron contents (*P* < 0.05). The maternal LF significantly promoted the iron stores in the heart, liver, spleen, and lung of piglets compared with Fe-Gly. (2) The maternal LF increased serum glutathione peroxidase (GSH-Px) and total superoxide dismutase (T-SOD) activities of sows. Compared with other groups, the total anti-oxidant capacity (T-AOC) activity of LF2 groups increased significantly (*P* < 0.05). (3) LF significantly increased piglet serum GSH-Px, T-SOD, and T-AOC activities (*P* < 0.05). (4) Gene expression levels of GSH-Px, and SOD in the duodenum and jejunum of the LF2 group were significantly higher than in the Fe-Gly group (*P* < 0.05), while the expression levels in the liver and heart were lower (*P* < 0.05). (5) The expression levels of hepcidin and LF in the liver and duodenum of the LF2 group were significantly higher than in the Fe-Gly group (*P* < 0.05). In conclusion, maternal LF supplementation showed remarkable effects on iron storage in neonatal piglets, and exhibited strong antioxidant activities, it is helpful to prevent the occurrence of iron deficiency, and improves the immune function of animals.

## Introduction

Iron is an essential element involved in various biochemical reactions ranging from the synthesis of hematopoiesis and the transport or exchange of oxygen, particularly to redox reactions (1–3). The iron nutritional status affects animals' immune function and growth performance (4, 5). A lack of iron may result in iron deficiency (ID) or iron deficiency anemia (IDA), which has adverse effects on health, often causing early mortality in piglets. Newborn piglets are particularly prone to ID due to their unique iron characteristics. In contrast to other animals, neonatal piglets possess inadequate iron storage, increase iron demand, and a deficiency of iron supply in breast milk. Numerous studies have focused on the efficacy of iron supplementation in pregnant sows, a lot of results showed the iron storage in piglets increased significantly with the level of the dietary glycine chelated and methionine iron in the gestational period and suckling period (6), the iron concentration in breast milk was also enhanced obviously (7). While some results found the maternal effects of dietary Fe-Gly were far lower for IDA prevention in piglets (8). At present, the most ferruginous feed additives in the swine diets were inadequate for the prevention of IDA in piglets. Therefore, it is of great significance to seek more efficient iron additives and investigate their physiological functions.

The study showed that Dietary iron supplements in pregnant sows had improved efficiencies on anemia symptoms, iron content in milk and embryo development. However, the maternal effect of the existing iron additives was not sufficient. Some studies found that the dietary glycine chelated during late pregnancy and lactation effectively prevented IDA in suckling piglets (6). Adding methionine iron to pregnant sows' diet increased the iron content in breast milk (7).

Lactoferrin (LF) is a multifunctional globular glycoprotein That widely exists in secretions, with the highest levels in milk and colostrum. LF is different from the natural glycoprotein polypeptide of the transferrin family. It is a non-heme iron-binding glycoprotein with binding and transporting functions for iron. Supplementing LF and its derivatives can promote the digestion and absorption of piglets, promote growth performance, mediate many essential physiological processes, regulate intestinal function, reduce weaning diarrhea in piglets, and positively impact the growth and health of pigs (9–11). Additionally, supplementation of dietary LF for sows during pregnancy and lactation can significantly increase milk secretion, pregnancy rate, and neonatal weight during lactation (12).

The Dahe black Pig is a famous endemic breed in Yunnan-Guizhou Plateau. These pigs have strong stress resistance, a high muscle fat content, and a lower water loss rate. However, the low survival rate of piglets limits the industrial production of the Dahe black Pig. This research aims to study the outcomes of maternal lactoferrin supplementation on iron contents, anti-oxidant parameters, and gene expression levels of anti-oxidant and Fe-acquisition in neonates, to support the protection and utilization of Dahe black Pig germplasm resources and understand the maternal mechanisms of LF.

## Materials and methods

### Animals

Sixty Dahe black sows (parity 3–4) with analogous body weights were randomly scattered into five groups, with 12 sows in each group. Each sow was kept in a single cage. These animals originated from a Dahe black Pigs Breeding Farm in Fuyuan county, Yunnan province. Disinfection, routine immunization, and sow management were required in exacting accordance with the requirements of pig farm management. Laboratory animals were raised and handled in rigorous accordance with the Institutional Animal Care and Use Committee of Yunnan Agricultural University.

### Diet preparation

The diets for sows are prepared according to the National Research Council (13) nutrient specifications for pregnant sows and the Feeding standard for lean-type pigs. The composition and nutrient levels of the basal diet are shown in Table 1, no iron was added to the basal diet. Pregnant sows were fed the experimental diets from 80 days after pregnancy. The diet was supplemented with 0 mg (control group), 100 mg (LF1 group), 200 mg (LF2 group), 300 mg (LF3 group) LF/kg, and 100 mg Fe/kg from ferric-glycine complex (Fe-Gly group), respectively. The LF used in this experiment was obtained from DMV International Co., Netherlands. The commercial name of the glycine-iron complex used in this experiment is Futiebao; the main components are iron-glycine complex (Fe-Gly), glycine ≥21%, and Fe^2+^ ≥17%. The iron-glycine complex was obtained from Zhejiang Huineng Animal Medicine Co., LTD. Pregnant sows were fed the experimental diets beginning 80 days before parturition.

**Table 1:** Composition and nutrient levels of the basal diet (air-dry basic).

**Ingredients**	**Content (%)**	**Nutrient levels^b^**	**Content**
Corn	67.00	DE(MJ/kg)	13.03
Soybean	20.00	CP	14.76
Wheat bran	9.00	Lys	0.88
Premix^a^	4.00	Met	0.25
Total	100	Thr	0.60
		Trp	0.17
		Ca	0.74
		AP	0.34
		Fe(mg/kg)	80.07

a Premix provided 8000 IU of vitamin A per kg of diet; Vitamin D3 1200 IU; Vitamin E 60 IU; Vitamin K3 2 mg; Vitamin B1 1 mg; Vitamin B2 4 mg; Vitamin B6 1 mg; Vitamin B12 17.00 ug; Folic acid 1.3 mg; D-biotin 0.2 mg; Pantothenic acid 12 mg; Choline 1 g chloride; Cu 12 mg, Mn 25 mg; With recent 100 mg; Se 0.30 mg; Iodine 0.4 mg.

b DE was a calculated value, while the others were measured values.

### Sample collection

After delivery, three placental samples were taken from the placenta of each sow and kept in a refrigerator at −80°C. Within 2–3 h after delivery, the breast was washed with disinfectant, wetting the hair near the breast. The first 3-4 expressions of breast milk were discarded, and then approximately ten mL of breast milk was collected in a sterilized test tube. Meanwhile, ten mL of blood (Venous blood collection used vacuum blood collection tube) was collected within 1 day and left for 1 h at room temperature to centrifuge serum at 1006.2×g for 15 min, with the serums then stored at −20°C. Blood samples from neonatal piglets were collected in the same way. Within 1 to 2 h after giving birth, three neonatal piglets of each sow were euthanized *via* a sodium pentobarbital overdose of 40 mg/kg body weight. Sample collection in liver, spleen, heart, and duodenum. The samples were frozen in liquid nitrogen and stored in a refrigerator at −80°C for quantitative Polymerase Chain Reaction (PCR) analysis. The second batch of samples was frozen and stored at −20°C.

### Analysis of samples

#### Blood analysis

Total-Superoxide Dismutase (T-SOD), Glutathione peroxidase (GSH-Px), Total antioxidant capacity (T-AOC), and Malondialdehyde (MDA) levels all were measured using the Kit (Nanjing Jiancheng Bioengineering lnstitute) according to the manufacturer's guidelines.

#### Iron content analysis of samples

According to GB/T 13885-2017, the samples were digested and prepared into solutions by dry digestion. The absorbance of each sample and blank solution were decisive using an atomic absorption spectrometer (AAS Vario 6, Analytik Jena AG).

#### Gene expression analysis

Quantitative real-time PCR (RT-qPCR) was performed using the iCyclerriQTM Real-Time PCR Detection System (BIO-RAD, USA) and Gradient PCR instrument (Eppendorf, Germany). Total RNA from the heart, liver, spleen, duodenum, and jejunum was extracted using the RNA simple Total RNA Kit (Takara, Japan), and cDNA was synthesized. The primers were synthesized by Sangon Biotech (Shanghai) Co. Ltd. Primer information is shown in Table 2. Use the β-actin gene as an internal reference gene.

**Table 2:** Information on primers used for RT-PCR.

**Gene**	**Primer sequence (5'-3')**	**Primer length/bp**	**Amplification length /bp**	**GenBank accession no**.
HEP	F:TCCGTTCTCCCATCCCAGAC	20	171	NC_007127.7
	R:GCAGCACATCCCACAGATTG	20		
LF	F:CAAATCTGACGAGTATCCA	19	186	NC_010455.5
	R:GGGGAAGACTACGGGTAA	18		
SOD	F:CAGGTCCTCACTTCAAT	18	254	NM_001190422.1
	R:CAAACGACTTCCAGCAT	17		
GSH-Px	F:AGAAGTGTGAGGTGAATGGC	20	325	NM_214201.1
	R:CCCGAGAGTAGCACTGTAAC	20		

### Statistical analysis

The data are presented as means and SEMs. Results were considered statistically significant at *P* ≤ 0.05. All statistical analyses were performed using the SPSS 21.0 software. A One-Way ANOVA in SPSS 21.0 was used for variance analysis, and the Duncan method was used for making multiple comparisons.

## Result

### The effects of LF on the serum, placenta and colostrum iron contents in sows

According to Table 3, LF supplementation improved the sows' serum, placental, and colostrum iron contents. The iron contents of serum and colostrum were the highest in the LF3 group (*P* < 0.05), and they decreased in turn in the LF2, LF1, and Fe-Gly groups. The Fe-Gly group had the highest placental iron content (*P* < 0.05), and LF2 had the second highest placental iron content (*P* < 0.05).

**Table 3:** Iron contents of the serum, placenta, and colostrum of sows.

**Items^1^, ^7^**	**Con^2^**	**LF1^3^**	**LF2^4^**	**LF3^5^**	**Fe-Gly^6^**	***P*-values**
Serum (mg/L)	21.34 ± 1.24^c^	23.18 ± 1.28^b^	23.46 ± 1.15^a, b^	25.01 ± 1.10^a^	22.06 ± 1.39^b, c^	<0.001
Placenta (μg /g)	21.05 ± 1.25^d^	21.76 ± 0.68^d^	32.08 ± 1.52^b^	30.10 ± 1.09^c^	58.61 ± 1.55^a^	<0.001
Colostrum (mg/L)	2.39 ± 0.14^d^	3.24 ± 0.05^c^	3.44 ± 0.09^b^	4.29 ± 0.16^a^	2.36 ± 0.15^d^	<0.001

1 n = 3: there were three replicates per treatment and four sows per replicates.

2 Con: the control group diet used the basal diet.

3 LF1: the LF1group diet supplemented with 100 mg LF/kg in the basal diet.

4 LF2: the LF2group diet supplemented with 200 mg LF/kg in the basal diet.

5 LF3: the LF3group diet supplemented with 300 mg LF/kg in the basal diet.

6 Fe-Gly: the Fe-Gly group diet supplemented with 100 mg Fe/kg from ferric-glycine complex in the basal diet.

7 Results are presented as mean ± SEM.

^ab^Within a row, values with different letter superscripts differ significantly (*P* < 0.05).

### Effect of different levels of LF on serum anti-oxidant indices in pregnant sows

According to Table 4, LF supplementation improved the serum T-AOC, T-SOD, and GSH-Px activities of sows but reduced the serum MDA activity. Compared with the Fe-Gly group, the activities of GSH-Px and T-SOD in LF2 and LF3 groups increased significantly (*P* < 0.05). T-AOC activity in the LF2 group was highest (*P* < 0.05), and decreased in turn in LF3, Fe-Gly, LF1, and control group, the control group was significantly lower than other groups (*P* < 0.05). The MDA content was the highest in the Fe-Gly group (*P* < 0.05), while the MDA contents in the LF groups were significantly lower than in the control and Fe-Gly groups (*P* < 0.05).

**Table 4:** Effects of different levels of LF on serum anti-oxidant indices of sows.

**Items^1^, ^7^**	**Con^2^**	**LF1^3^**	**LF2^4^**	**LF3^5^**	**Fe-Gly^6^**	***P*-values**
GSH-Px (U/mL)	135.42 ± 6.29^c^	141.81 ± 4.95^b^	154.20 ± 5.08^a^	153.59 ± 4.58^a^	142.04 ± 6.58^b^	<0.001
T-SOD (U/mL)	90.79 ± 1.86^c^	100.37 ± 3.86^b^	103.80 ± 2.55^a^	104.65 ± 1.48^a^	93.39 ± 2.61^c^	<0.001
T-AOC (U/mL)	14.42 ± 0.87^d^	15.55 ± 0.65^d^	28.01 ± 2.64^a^	24.97 ± 1.68^b^	22.64 ± 1.37^c^	<0.001
MDA (nmol/mL)	7.61 ± 0.22^b^	3.32 ± 0.14^e^	4.45 ± 0.17^d^	6.91 ± 0.17^c^	12.63 ± 0.27^a^	<0.001

1 n = 3: there were three replicates per treatment and four sows per replicates.

2 Con: the control group diet used the basal diet.

3 LF1: the LF1group diet supplemented with 100 mg LF/kg in the basal diet.

4 LF2: the LF2group diet supplemented with 200 mg LF/kg in the basal diet.

5 LF3: the LF3group diet supplemented with 300 mg LF/kg in the basal diet.

6 Fe-Gly: the Fe-Gly group diet supplemented with 100 mg Fe/kg from ferric-glycine complex in the basal diet.

7 Results are presented as mean ± SEM.

^ab^Within a row, values with different letter superscripts differ significantly (*P* < 0.05).

### The effects of LF on the serum and tissue iron contents of neonatal piglets

From the data in Table 5, in the tissues of piglets, the iron content in the heart, lung, duodenum, and jejunum was the highest in the control group (*P* < 0.05). However, the serum iron content in the control group was not the highest, the highest was LF3 group (*P* < 0.05). In the experimental group. The iron content in the heart, liver, spleen, and lung in the three LF groups was higher than that of the Fe-Gly group (*P* < 0.05). The addition of lactoferrin or ferrous glycine significantly reduced the accumulation of iron in piglets' intestines (*P* < 0.05).

**Table 5:** Iron contents in the serum and tissues of neonatal piglets (μg/g).

**Items^1^, ^7^**	**Con^2^**	**LF1^3^**	**LF2^4^**	**LF3^5^**	**Fe-Gly^6^**	***P*-values**
Heart	47.89 ± 1.01^a^	24.65 ± 1.22^c^	25.86 ± 0.78^c^	32.65 ± 1.07^b^	18.78 ± 0.52^d^	<0.001
Liver	51.53 ± 1.22^b^	44.91 ± 0.94^d^	47.68 ± 1.19^c^	55.10 ± 0.73^a^	38.26 ± 0.81^e^	0.013
Spleen	68.60 ± 1.95^c^	56.36 ± 1.96^d^	72.51 ± 1.49^b^	73.68 ± 1.98^a^	55.02 ± 1.64^e^	0.007
Lung	47.01 ± 1.83^a^	27.36 ± 1.38^d^	29.89 ± 1.16^c^	34.24 ± 0.95^b^	24.80 ± 0.69^e^	<0.001
Duodenum	53.58 ± 1.42^a^	16.75 ± 1.11^e^	18.73 ± 1.02^d^	21.31 ± 0.54^c^	22.64 ± 0.63^b^	<0.001
Jejunum	53.76 ± 0.43^a^	15.41 ± 0.59^d^	16.86 ± 0.76^c^	18.96 ± 0.99^b^	17.48 ± 0.89^c^	<0.001
Serum (mg/L)	21.58 ± 0.77^b^	17.57 ± 0.97 ^d^	20.22 ± 0.96^c^	33.92 ± 0.93^a^	21.28 ± 0.96^b^	<0.001

1 n = 3: there were three replicates per treatment and twelve neonatal piglets per replicates.

2 Con: the control group diet used the basal diet.

3 LF1: the LF1group diet supplemented with 100 mg LF/kg in the basal diet.

4 LF2: the LF2group diet supplemented with 200 mg LF/kg in the basal diet.

5 LF3: the LF3group diet supplemented with 300 mg LF/kg in the basal diet.

6 Fe-Gly: the Fe-Gly group diet supplemented with 100 mg Fe/kg from ferric-glycine complex in the basal diet.

7 Results are presented as mean ± SEM.

^ab^Within a row, values with different letter superscripts differ significantly (*P* < 0.05).

### Effects of different levels of LF on the serum anti-oxidant indices of neonatal piglets

According to Table 6, LF supplementation improved neonatal piglets' serum T-AOC, GSH-Px, and T-SOD activities (*P* < 0.05) but reduced the MDA activity (*P* < 0.05), which was consistent with the changing of sows. The changing trend of the Fe-Gly group and LF group was the same, but the activities of various enzymes in the LF group were significantly increased with the increase of Fe content in LF. The activities of T-AOC and MDA were positively correlated with the amount of lactoferrin added. The addition of lactoferrin greatly improved the activity of T-AOC. In addition, compared with the control group, MDA activity in the LF groups and the Fe-Gly group decreased significantly (*P* < 0.05).

**Table 6:** Effects of different levels of LF on the serum anti-oxidant indices of neonatal piglets.

**Items^1^, ^7^**	**Con^2^**	**LF1^3^**	**LF2^4^**	**LF3^5^**	**Fe-Gly^6^**	***P*-values**
GSH-Px (U/mL)	139.17 ± 2.70^b^	155.51 ± 4.14^a^	150.79 ± 4.81^a^	136.81 ± 5.67^b^	132.89 ± 5.10^b^	<0.001
T-SOD (U/mL)	49.48 ± 0.92^d^	63.77 ± 1.09^a^	58.86 ± 2.20^b^	56.21 ± 1.60^c^	58.87 ± 2.46^b^	<0.001
T-AOC (U/mL)	5.60 ± 0.18^e^	16.49 ± 0.45^c^	35.70 ± 0.90^b^	37.92 ± 1.37^a^	6.79 ± 0.08^d^	<0.001
MDA (nmol/mL)	28.40 ± 1.63^a^	16.35 ± 1.12^b^	15.14 ± 0.58^c^	17.32 ± 0.87^b^	13.25 ± 0.42^d^	<0.001

1 n = 3: there were three replicates per treatment and twelve neonatal piglets per replicates.

2 Con: the control group diet used the basal diet.

3 ^3^LF1: the LF1group diet supplemented with 100 mg LF/kg in the basal diet.

4 ^4^LF2: the LF2group diet supplemented with 200 mg LF/kg in the basal diet.

5 ^5^LF3: the LF3group diet supplemented with 300 mg LF/kg in the basal diet.

6 ^6^Fe-Gly: the Fe-Gly group diet supplemented with 100 mg Fe/kg from ferric-glycine complex in the basal diet.

7 ^7^Results are presented as mean ± SEM.

^ab^Within a row, values with different letter superscripts differ significantly (*P* < 0.05).

### Effects of different levels of LF on anti-oxidant gene expression in the tissues of neonatal piglets

#### Expression of GSH-Px gene in neonatal piglets

**Figure 1:**
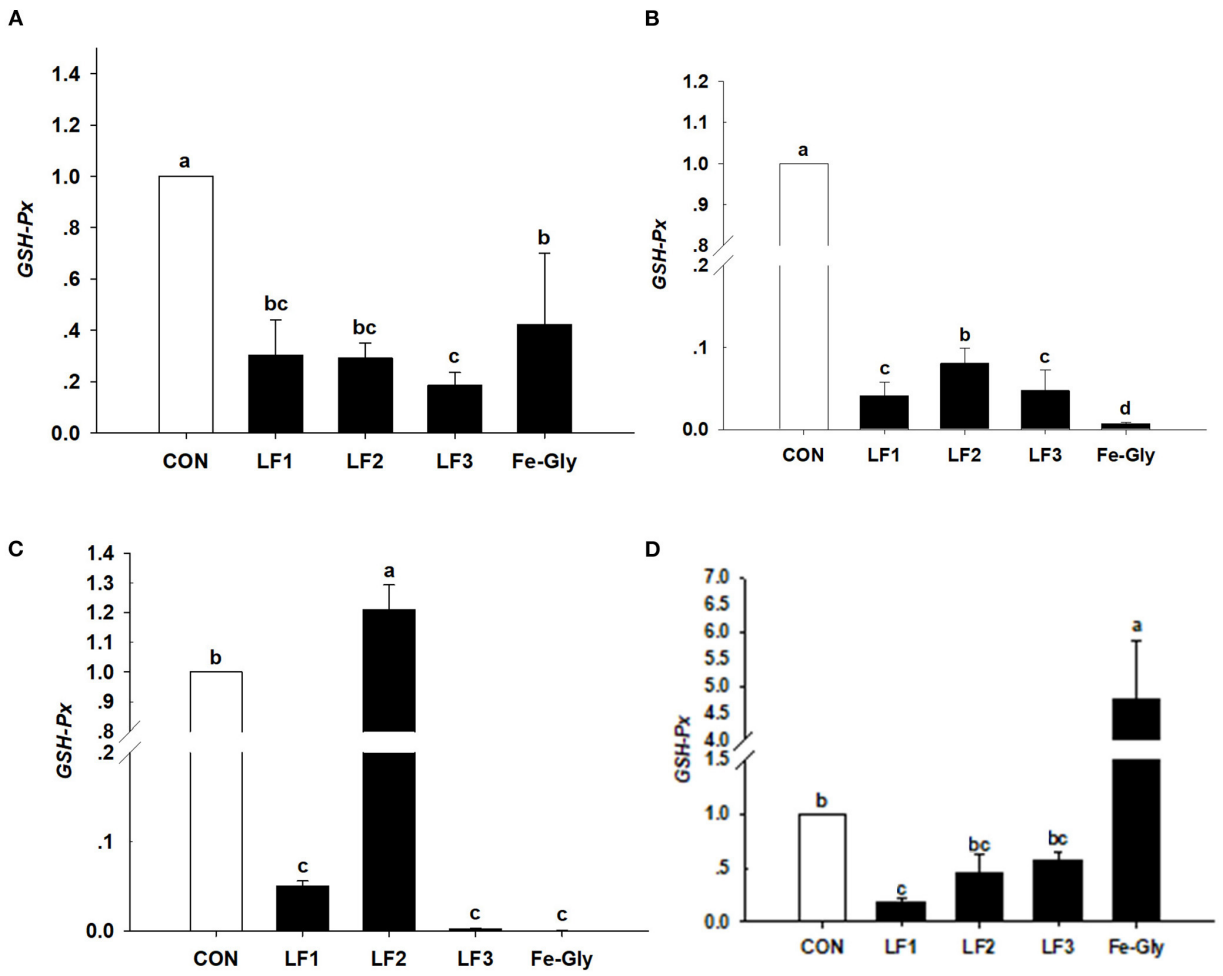
Effects of different levels of LF on the expression of the GSH-Px gene in liver **(A)**, duodenum **(B)**, jejunum **(C)**, and heart **(D)** of neonatal piglets. Con: the control group diet used the basal diet. LF1: the LF1group diet supplemented with 100 mg LF/kg in the basal diet. LF2: the LF2group diet supplemented with 200 mg LF/kg in the basal diet. LF3: the LF3group diet supplemented with 300 mg LF/kg in the basal diet. Fe-Gly: the Fe-Gly group diet supplemented with 100 mg Fe/kg from ferric-glycine complex in the basal diet. ^ab^Within a row, values with different letter superscripts differ significantly (*P* < 0.05).

As shown in Figure 1, the expression levels of the GSH-Px gene in the liver, duodenum, and heart of the LF and Fe-Gly groups were significantly lower than that in the control group (*P* < 0.05). The expression level of the GSH-Px gene in the jejunum of the LF2 group was significantly higher than those of the LF1, LF3, and Fe-Gly groups (*P* < 0.05). The Fe-Gly group had higher expression of the GSH-Px gene in the heart than the LF groups (*P* < 0.05).

#### Expression of the SOD gene in neonatal piglets

**Figure 2:**
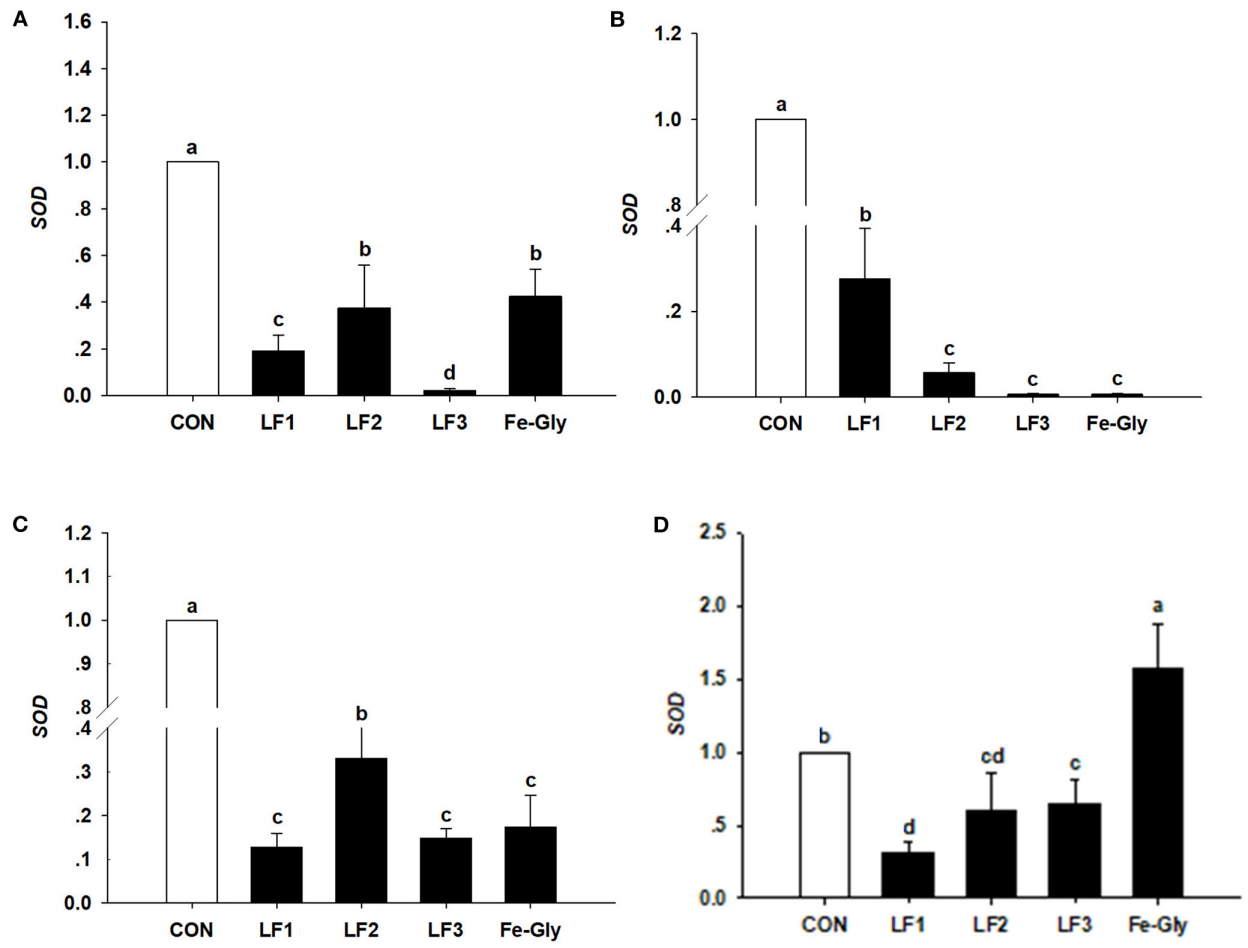
The expression levels of the SOD gene in liver **(A)**, duodenum **(B)**, jejunum **(C)**, and heart **(D)** of neonatal piglets. Con: the control group diet used the basal diet. LF1: the LF1group diet supplemented with 100 mg LF/kg in the basal diet. LF2: the LF2group diet supplemented with 200 mg LF/kg in the basal diet. LF3: the LF3group diet supplemented with 300 mg LF/kg in the basal diet. Fe-Gly: the Fe-Gly group diet supplemented with 100 mg Fe/kg from ferric-glycine complex in the basal diet. ^ab^Within a row, values with different letter superscripts differ significantly (*P* < 0.05).

The data in Figure 2 demonstrate that the expression levels of the SOD gene in the liver, jejunum, and duodenum were significantly downgraded by LF and Fe-Gly (*P* < 0.05). In contrast, the expression in the heart of the Fe-Gly group was significantly increased (*P* < 0.05). SOD expression levels in the liver and jejunum of the LF2 group were significantly higher than in the LF1 (*P* < 0.05) and LF3 (*P* < 0.05) groups.

### Effects of different levels of LF on the expression of the ferritin gene in the tissues of neonatal piglets

#### Expression of hepcidin gene in the tissues of neonatal piglets

**Figure 3:**
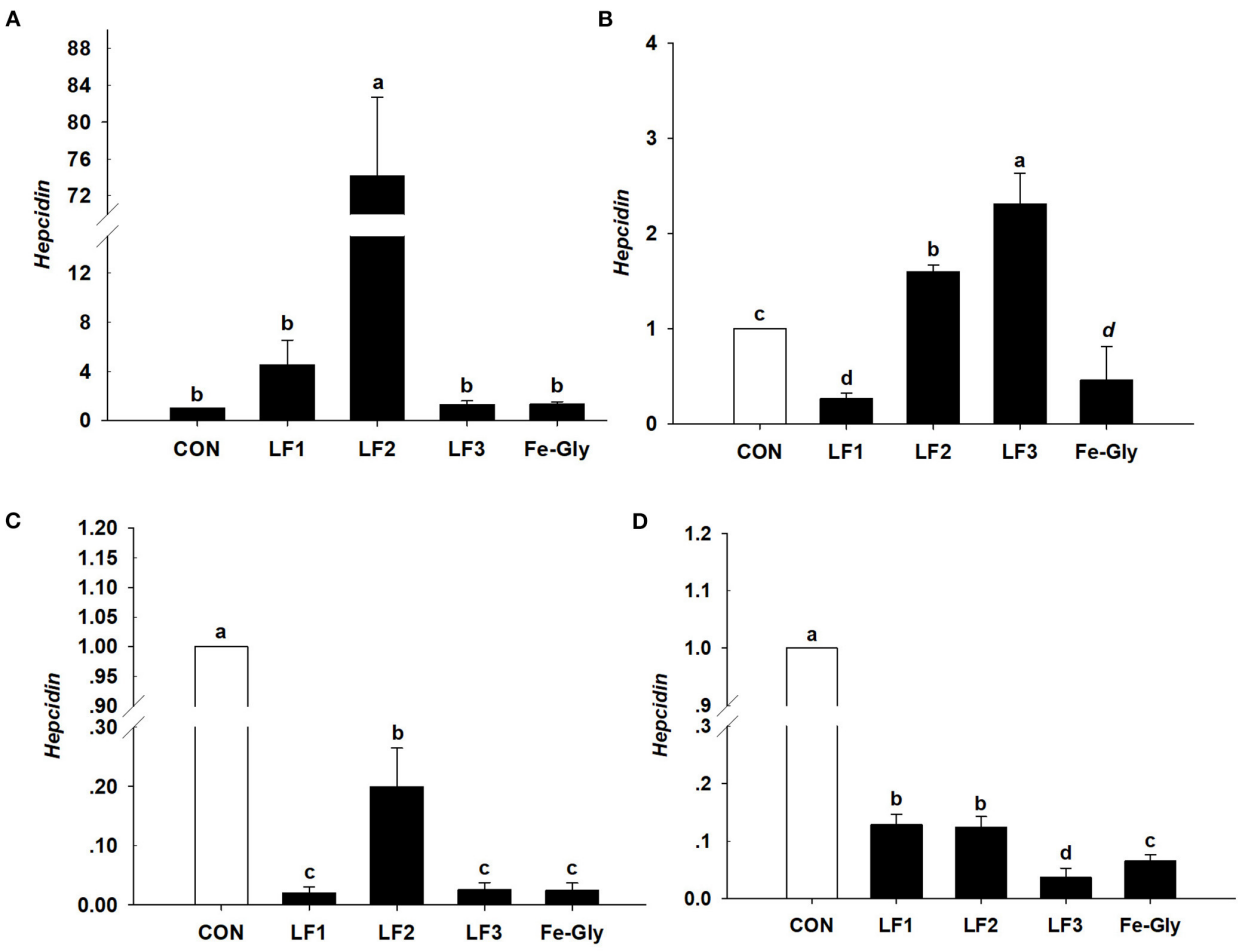
The expression levels of the hepcidin gene in liver **(A)**, spleen **(B)**, duodenum **(C)**, and jejunum **(D)** of neonatal piglets. Con: the control group diet used the basal diet. LF1: the LF1group diet supplemented with 100 mg LF/kg in the basal diet. LF2: the LF2group diet supplemented with 200 mg LF/kg in the basal diet. LF3: the LF3group diet supplemented with 300 mg LF/kg in the basal diet. Fe-Gly: the Fe-Gly group diet supplemented with 100 mg Fe/kg from ferric-glycine complex in the basal diet. ^ab^Within a row, values with different letter superscripts differ significantly (*P* < 0.05).

As shown in Figure 3, the hepcidin gene was detected in the liver, spleen, duodenum, and jejunum. The expression level of hepcidin in the liver of the LF2 group was significantly higher than those of the other groups (*P* < 0.05). Hepcidin expression levels in the spleens of the LF2 and LF3 groups were significantly higher than those in the other groups (*P* < 0.05). LF and Fe-Gly downregulated hepcidin expression in the duodenum and jejunum (*P* < 0.05).

#### Expression of the LF gene in various tissues of neonatal piglets

**Figure 4:**
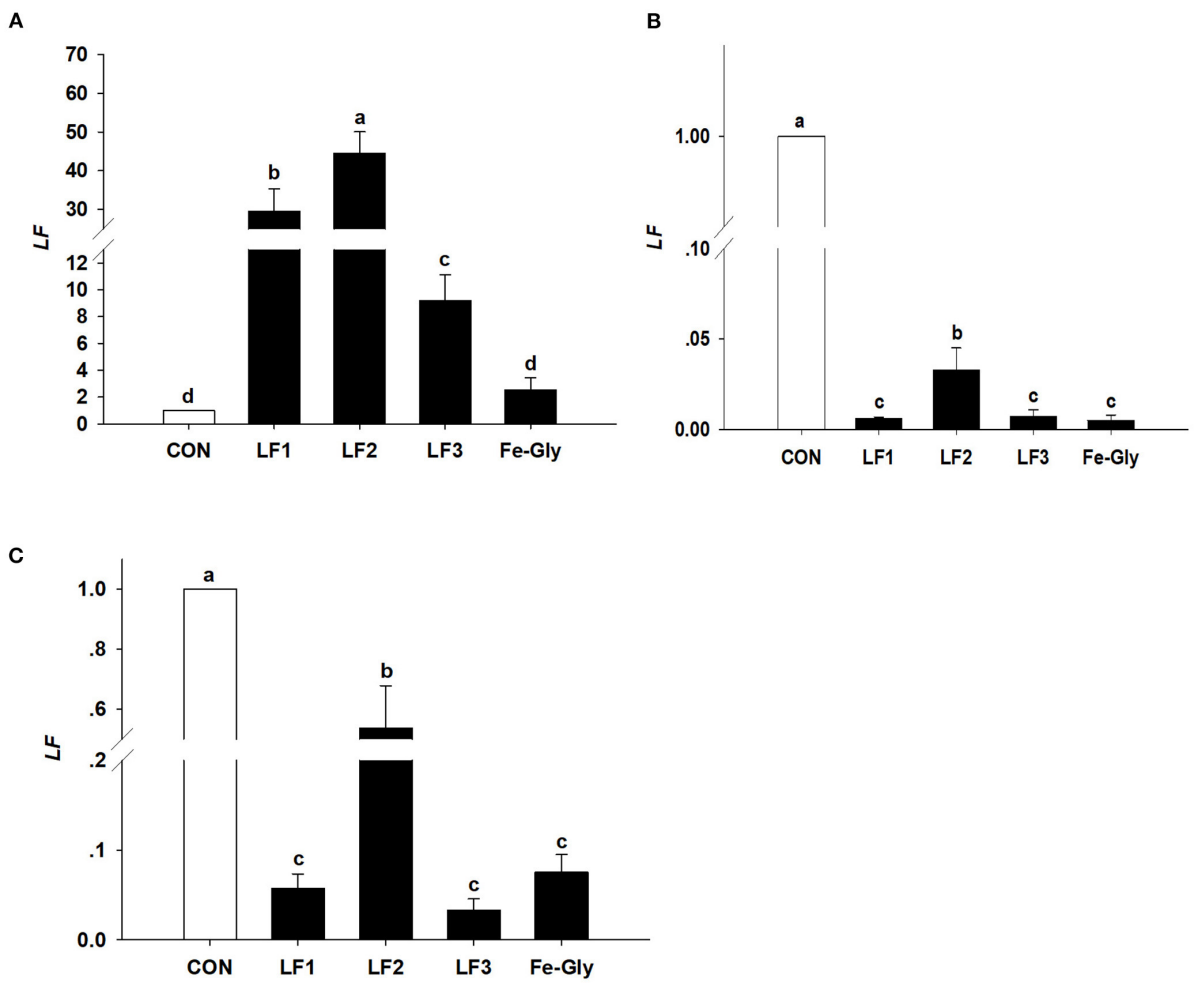
The expression levels of the LF gene in liver **(A)**, duodenum **(B)**, and jejunum **(C)** of neonatal piglets. Con: the control group diet used the basal diet. LF1: the LF1group diet supplemented with 100 mg LF/kg in the basal diet. LF2: the LF2group diet supplemented with 200 mg LF/kg in the basal diet. LF3: the LF3group diet supplemented with 300 mg LF/kg in the basal diet. Fe-Gly: the Fe-Gly group diet supplemented with 100 mg Fe/kg from ferric-glycine complex in the basal diet. ^ab^Within a row, values with different letter superscripts differ significantly (*P* < 0.05).

As seen in Figure 4, the LF gene was detected in the liver, duodenum, and jejunum. The LF gene expression level in the liver of the LF2 group was significantly higher than those in the control and Fe-Gly groups (*P* < 0.05). LF gene expression levels in the duodenum, and jejunum of neonatal piglets were significantly downregulated by dietary LF and Fe-Gly supplementation of sows (*P* < 0.05), and the expression level of the LF2 group was significantly higher than those of the LF1, LF3 and Fe-Gly groups (*P* < 0.05).

## Discussion

### Effects of different levels of LF on iron contents in placental, serum, colostrum of saws, and tissues of piglets

Iron is one of the vital nutrition elements, especially for pregnant animals and infants (4, 5). During pregnancy and lactation, large amounts of iron stores are transported from mother to fetus, with lactation meeting the increasing demands of the fetus. Multi-fetal animals consumed even more maternal iron especially sows with a high farrowing rate. The study showed that the amount of iron stored in sows gradually decreases and remains at a lower level after their third fetus (14). For many decades, the majority of previous research has focused on the effect of iron additive composition, and additive concentration on pregnant sows (15–17), but only a few were related to fetal iron levels (8). Dong et al. (16, 17) reported that dietary iron amino acid chelated improved iron storage in pregnant sows, and the addition of glycine chelated iron significantly increased the iron content in the placenta, colostrum, and conventional milk.

As new iron additive, the characteristics, biological function, and application of LF were studied in the articles. Jahan et al. (12) reported LF could improve the milk yield, litter sizes, and serum immunoglobulin levels of pregnant sows. Our previous research (18) found adding LF to the diets of sows during late pregnancy significantly improved their reproductive performance. In this experiment, the serum iron concentration of piglets in the LF and Fe-Gly supplemented groups were significantly increased, and the effect of Fe-Gly supplementation was less than the effects of LF supplementation. LF particularly showed a significant promotion of iron levels in the placental and the colostrum, the accumulating amount of iron in the placenta was 94.72% in the Fe-Gly group compared with the LF3 group, while the iron concentration in the colostrum of the Fe-Gly group was lower than 81.78% contrast against the LF3 group. These results were similar to the report of Nie (15), the dietary hydrolyzed soybean protein chelated iron significantly increased the iron content in milk. Xu et al. (19) reported that the addition of 60 mg/kg of methionine iron improved iron levels in the placenta, breast milk, and piglets. Breast milk was the main iron source in the early stage of neonates, it was critical in improving the iron concentration in the colostrum, while Fe-Gly as a widely used iron additive was not very effective in it (20).

**Figure 5:**
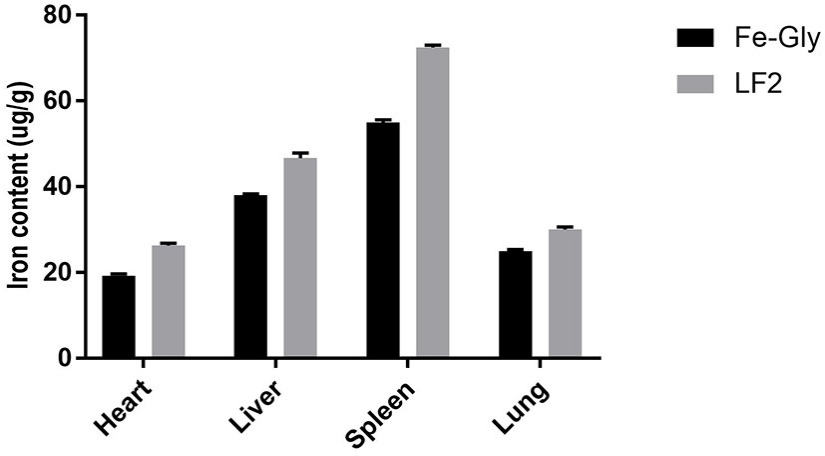
Effects of lactoferrin and Fe-Gly on the iron content in herat, liver, spleen, and lung. LF2: the LF2group diet supplemented with 200 mg LF/kg in the basal diet. Fe-Gly: the Fe-Gly group diet supplemented with 100 mg Fe/kg from ferric-glycine complex in the basal diet.

**Figure 6:**
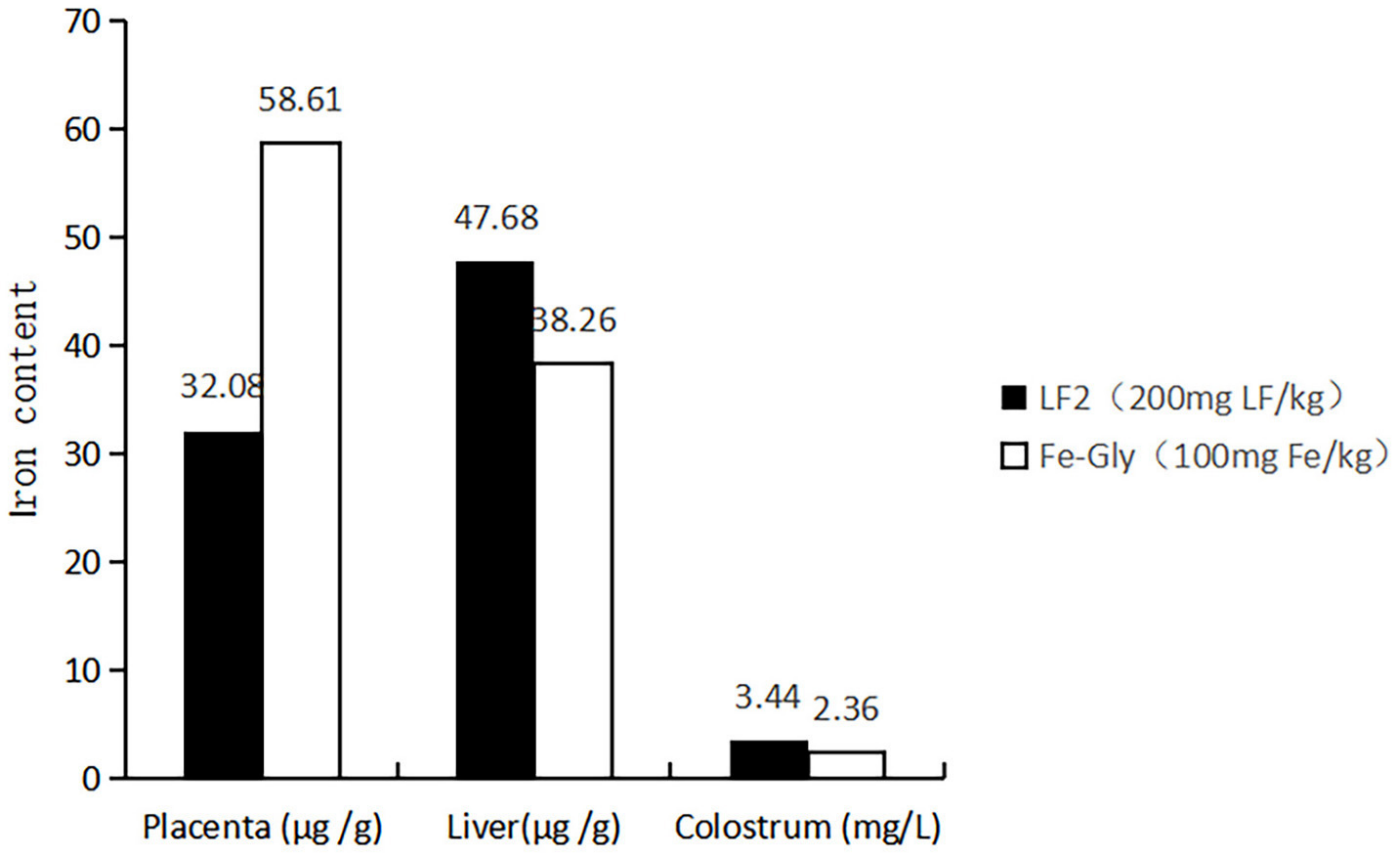
Effects of lactoferrin and Fe-Gly on the iron content in placenta, liver, and colostrum. LF2: the LF2group diet supplemented with 200 mg LF/kg in the basal diet. Fe-Gly: the Fe-Gly group diet supplemented with 100 mg Fe/kg from ferric-glycine complex in the basal diet.

Maternal nutrition affects the growth of their descendants. The amino acid chelate iron, particularly Fe-Gly, is a widely used iron additive. However, maternal efficacy and reliability are inconsistent. Many studies have investigated the effects of maternal supplementation of iron on iron levels in fetuses (21), while the maternal efficacy and reliability are inconsistent. Xu et al. (7) and Wang et al. (6) found that adding glycine chelated iron to the sow's diet in late pregnancy and lactation could offer enough iron to meet growth and prevent IDA in suckling piglets. Xu et al. (19) reported that the addition of 60 mg/kg methionine iron to the sows' diet improved iron levels in the placenta and breast milk of piglets. The addition of 0.2% iron-supplemented probiotics (mainly composed of iron-enriched yeast, ferrous sulfate, and lactic acid bacteria) could increase the iron levels in sows and piglets (22), and the digestibility and availability of iron in the fetus were lower (23, 24). Shi (25) reported that dietary amino acid chelated iron enhanced iron storage in piglets, but the effects were not beneficial to sows. Similar reports by Zeng et al. (26) showed that 120 mg/kg amino acid iron complex in the diet of pregnant sows improved the iron nutrition status of sows and newborn piglets, but the efficiency in piglets was inferior to sows. LF has a better intestinal passage and higher utilization compared with amino acid iron complexes and ferrous sulfate [by Shi (25), Zeng et al. (26)]. The maternal effects of LF on iron storage in the fetus were emphatically studied in the paper. According to the results, the LF supplementation showed increased effects on the iron content in the tissue of piglets compared to Fe-Gly. According to Figure 5, the iron content in the viscera of piglets in the LF2 group and Fe-Gly group showed a similar trend, and the iron content in the viscera of the LF2 group was higher than that of the Fe-Gly group. As shown in Figure 6, although the placenta iron content of sows in the Fe-Gly group is higher than that in the LF2 group the influence of lactoferrin on piglets' internal organs is still greater than that of glycine iron. This mean the LF supplementation exerted strong effects on iron storage in organs and tissues of neonates, the effects of Fe-Gly were far less than those of the LF. The finding suggested that LF could effectively penetrate the placental barrier, and the mechanisms might be closely related to the transplacental iron transport or mammary transport of iron-containing protein. The efficacy and effective delivery of iron supplementation required further research.

### Effects of LF on serum anti-oxidant enzyme activities of sow and neonates

Elemental iron participates in electron transportation and the anti-oxidative system as a component of cytochrome oxidase. In oxidative stress, the excessive reactive oxygen species (ROS) and reactive nitrogen species (RNS) may damage the anti-oxidant defense system *in vivo*. To maintain the homeostasis of radicals in cells, the activity of anti-oxidant enzymes increased. Previous studies have reported that LF can improve anti-oxidant capacity in the body or tissues. Another study found that the generation of OH, superoxide radicals, and DPPH radicals could be inhibited by recombinant human LF and bovine LF (27). Our previous study found the addition of 250 mg/kg and 500 mg/kg LF significantly increased piglet serum NO levels (28). LF down-regulated ROS levels by triggering the expression of SOD, GSH-Px, and catalase (29). Based on previous research, this study focused on the maternal effects of LF and Fe-Gly on anti-oxidant enzymes in the serum of sows and newborn piglets. The study showed both LF and Fe-Gly enhanced the activities of GSH-Px, T-AOC, and T-SOD while decreasing serum MDA levels of sows. Additionally, maternal supplementation of LF enhanced the activities of GSH-Px, T-AOC, and T-SOD and decreased the serum MDA levels of newborn piglets. Compared with Fe-Gly, the effect of LF was even more significant in sows and neonates. Our research results are similar to those of other scholars (30–32). We infer that the mechanism might be related to the structural characteristics in which LF binding ferric ions, and reduces the formation of free radicals. The experimental conditions of this study confirmed that supplemented with 200 mg/kg LF has the best outcomes for sows, and adding 300 mg/kg was the best for neonates.

### Effects of maternal LF on the expression of anti-oxidant genes and genes in iron transport systems in the tissues of neonates

The addition of lactoferrin also affected the expression level of antioxidant genes in animals. The research of Wang et al. showed that the bovine lactoferrin (bLf) significantly increased anti-oxidant enzyme activity and gene expression levels in piglets, the genetic expression of CuZnSOD and catalase (CAT) in the longissimus dorsi muscle of piglets were significantly promoted with the increasing of dietary bLf (33). It has also been reported that LF promoted the genetic expression of proteolytic enzyme and amino acid transporter (CAT 1) in the jejunum of piglets (34). The previous studies from our laboratory reported that LF significantly increased the β-defensin gene expression in the tissues of sows and piglets (28, 35). The research of Safaeian showed that LF was effective in depressing intracellular and extracellular hydrogen peroxide. When human umbilical vein endothelial cells were pretreated with LF for 24 h and exposed to 0.5 Mm H_2_O_2_ for 2 h, the concentration of H_2_O_2_ was observed to decrease while ferric/antioxidant genes activity increased (36).

In order to investigate the antioxidant mechanism and the mechanisms of LF management, in this study, anti-oxidant genes and iron transport systems genes in the tissues of neonates were analyzed. The research showed that the expression levels of GSH-Px and SOD in the duodenum and jejunum of the LF2 group were significantly higher than in the Fe-Gly group, while the expression levels in the liver and heart were lower. The results indicated that LF improves the antioxidative function of the intestinal tract. These results were similar to the reports of Safaeian et al. and Pu (36, 37). The expression levels of hepcidin and LF in the liver and duodenum of the LF2 group were significantly higher than in the Fe-Gly group. However, compared with the control group, the expression levels of GSH-Px, SOD, hepcidin, and LF gene in the liver, duodenum, and intestine of neonates were significantly decreased by LF or Fe-Gly. LF and hepcidin are members of the BMP/SMAD signal transduction pathway involved in the mechanism of Fe iron. The expression of hepcidin mRNA directly reflects the nutritional status of iron *in vivo*, and its abnormal expression would lead to a disorder involving iron metabolism and absorption. We speculate that the decrease in the expression levels of hepcidin and LF genes in the LF2 and Fe-Gly groups may be due to the fact that the demand for iron in tissues is sufficient, and further research is needed. The current study demonstrated that dietary LF supplementation of sows significantly affects the neonatal anti-oxidant capacity of piglets, and the best effect was obtained when the amount of LF was 200 mg/kg.

## Conclusions

Maternal LF supplementation showed remarkable effects on iron storage in neonatal piglets, and exhibited strong antioxidant activities. The supplementation of LF is helpful to prevent the occurrence of IDA in piglets and improve antioxidant activity.

## Data availability statement

The original contributions presented in the study are included in the article/supplementary material, further inquiries can be directed to the corresponding author.

## Ethics statement

The animal study was reviewed and approved by Institutional Animal Care and Use Committee of Yunnan Agricultural University.

## Author contributions

CZ and QA contributed to the conception of the study. XX and PJ conducted the animal feeding experiment and sample collection. CL and HP performed the data analyses and wrote the manuscript. RG and ML helped perform the analysis with constructive discussions. All authors contributed to the article and approved the submitted version.

## Funding

This study was supported by National Natural Science Foundation of China (No. 32060758).

## Conflict of interest

The authors declare that the research was conducted in the absence of any commercial or financial relationships that could be construed as a potential conflict of interest.

## Publisher's note

All claims expressed in this article are solely those of the authors and do not necessarily represent those of their affiliated organizations, or those of the publisher, the editors and the reviewers. Any product that may be evaluated in this article, or claim that may be made by its manufacturer, is not guaranteed or endorsed by the publisher.
